# Can scope-of-practice transfer psychiatrists be up for their job? A cross-sectional study of clinical competence status and related factors

**DOI:** 10.1186/s12909-022-03860-3

**Published:** 2022-11-10

**Authors:** Huan Wang, Zuowei Li, Ruoxin Fan, Shaoli Tian, Xianmei Yang

**Affiliations:** 1 Nursing Department, the Third Hospital of Mianyang ▪ Sichuan Mental Health Centre, Mianyang, Sichuan Province 621000 People’s Republic of China; 2 Department for prevention and control of mental illness, the Third Hospital of Mianyang ▪ Sichuan Mental Health Centre, Mianyang, Sichuan Province 621000 People’s Republic of China

**Keywords:** Clinical competence, Continuing medical education, Psychiatrist, China

## Abstract

**Background:**

The great economic and social changes have resulted in the prevalence of mental disorder increasing year by year in China. Mental health medical service resources of China are significantly insufficient. The program of Transfer Training for psychiatrist was launched in China in October 2015. Thousands of physicians completed the transfer training and obtained certificates. To date, there is little evidence to identify the status and related factors of clinical competence among scope-of-practice transfer psychiatrists in China.

**Purpose:**

This study aimed to investigate the status and related factors of clinical competence among scope-of-practice transfer psychiatrists of Sichuan Province, China.

**Patients and methods:**

The sample was composed of 291 physicians who certificated the transfer training. Data were collected between September and November 2021, using self-made questionnaire with a total of 22 items to record demographic characteristics, practice status and workplace of participants. Descriptive statistics analysis, independent sample T-test, one-way ANOVA, Spearman rank correlation, and multiple regression analysis were used to analyze the data.

**Results:**

The clinical competence of participants score was (8.02 ± 1.48). Significant differences were found in clinical competence scores among: the subgroups of practice category, reasons for attending in the transfer training for psychiatrists, whether transfer to/ add mental health practice registration, whether engage in mental / psychological work after training, whether the level of transfer training meeting participants’ job needs, whether the level of transfer training meeting their theoretical learning needs, whether the level of transfer training meeting their clinical practice needs, salary change compared with pre-training, whether join in continuing education after training, whether wanted to join in continuing education after training, whether the workplace before training has mental / psychological department, whether the workplace after training has mental / psychological department, institutional nature, institutional level and institutional affiliation. Multiple regression analysis identified that level of transfer training meeting participants’ job needs, level of transfer training meeting their clinical practice needs, Whether the workplace before training has mental / psychological department, whether wanted to join in continuing education after training, institutional nature were the contributors of clinical competence.

**Conclusion:**

The study demonstrated that clinical competence of scope-of-practice transfer psychiatrists needed to be improved. Whether workplace has mental/psychological departments was an important factor of clinical competence. Besides, interest of physicians is another crucial factor for their clinical competence. The continuing education of those psychiatrists may be one effective measure considering their factual working conditions.

## Introduction

Over the past few decades, China has undergone tremendous economic and social changes. These changes are likely to bring about a general increase of psychological pressure and stress, resulting in the prevalence of mental disorder increasing year by year. China Mental Health Survey (CMHS) investigated the prevalence of mental disorders in 2019, covering a total of 32,552 people distributed in 31 provinces, 157 counties or districts. The results showed the lifetime prevalence of mental disorders was 16.6% [1]. Though this lifetime prevalence was slightly lower than that of European, the United State and other areas ranging between 14 and 26% [2–5]. If considering total population - 1.4 billion, this prevalence still indicates that a very large number of individuals are affected by mental disorders in China [1].

There is a huge demand for mental health services in China. Now mental health medical service resources of China are significantly insufficient, gap still exists between medical supply and the increasing demand of individuals affected by mental disorders. By the end of 2015, there were 2936 facilities providing mental health services, 433,090 psychiatric beds in total all cross China. The number of psychiatrists is 2.19 per 100,000 population [6], higher than global averages (1.7 per 100,000 population), but roughly 4 times less than that in high-income countries (8.6 per 100,000 population) reported by the World Health Organization in 2020 [7]. Sichuan province, the investigate site of current study, has a total population of 91 million. As of 2014, number of psychiatrists in Sichuan province is 2.18 per 100,000 population. Mental health service in Sichuan province was insufficient and was distributed extreme unevenly among different areas, especially in Ganzi Prefecture and Aba Prefecture which had a blank of psychiatrists and psychiatric beds [8].

The National Health Commission of People’s Republic of China proposed the Implementation Plan of the Transfer Training for psychiatrists Program in October 2015, to develop more qualified psychiatrists and improve mental health service ability. This program, a kind of continuing medical education, is to convert one non-psychiatrist to a qualified psychiatrist by standardized training and evaluation. The physicians should learn theoretical knowledge and clinical skills of psychiatry and clinical psychology. The training time is no less than 12 months consisting of 1-month theoretical study, 10-months clinical practice and 1-month community practice [9]. Previous study [10] showed this program alleviated the shortage of mental health professionals in the primary care and remote areas effectively, promoted the construction of mental health service.

Until now, several provinces have held the transfer training for psychiatrist program. Sichuan province launched the first this program in May 2016. By September 2020, 468 physicians completed the transfer training and obtained certificates, 87 physicians were still at training. Since the program began, the number of counties without psychiatrists has decreased from 85 to 15 within a total of 183 counties in Sichuan province.

The successful completion of this training should provide physicians with basic level of knowledge and skills to carry out a psychiatrist’s duties at a hospital. However, shortage of training duration and lack of fundamental psychological knowledge lead to a debate of the competency among those transferred psychiatrists. Clinical competence refers to knowledge, skills, abilities, and traits of physicians to complete clinical work. In fact, most psychiatrists who finished the training demonstrated a strong demand of further learning to better adapt to their clinic.

At present, there were several studies related with scope-of-practice transfer psychiatrists sharing training experiences, training modes and how to improve theoretical training quality. Little research has been conducted to investigate the post-training practice status and work capacity of scope-of-practice transfer psychiatrists [11–13]. Given the above-mentioned considerations, the aim of the present cross-sectional study was to measure the clinical competence and to explore its related factors of physicians who completed training in Sichuan province, China.

## Material and methods

### Study design and participants

It was a cross-sectional study, conducted in November 2021. Participants were recruited from all students certificated in trainings from 2016 to 2021 by convenience sampling. The inclusion criteria were as follows: 1) Students who completed training (including theoretical training1 month, clinical practice 10 month and community practice 1 month) and obtained training certificate; 2). Be willing to participate. People who didn’t engage in medical industry or had already retired (male> 60 years old, female > 55 years old) were excluded from the study.

A total of 293 questionnaires were collected, two of them were discarded due to missing data on the major variables. Two hundred ninety-one participants were included in the study finally, the effective ratio was 99.23%.

### Data collection

All data were collected through questionnaire survey. E-questionnaire was created through Questionnaire Star. Researchers filled out the e-questionnaire firstly and modified something. The e-questionnaires were sent to participants through WeChat group consisting of all students. All participants read and understood the study purpose, procedures, potential risks and benefits, and guaranteed confidentiality and voluntary participation before data collection. Each participant provided informed consent after they agreed to participate in this study. Time needed to complete the survey was 20 to 30 minutes. Researchers checked the completed questionnaires through data base of Questionnaire Star, questionnaires with obvious omissions or logic errors would be removed.

### Measures

#### Demographics

Demographic characteristics included gender, age, practice level, practice category, professional ranks and titles, and reasons for attending in the transfer training for psychiatrists. Practice level included practicing physician and physician assistant. Practice category was divided into clinical medicine and traditional Chinese medicine. Professional ranks and titles ranked from resident physician to associate chief physician and above. Another single item was used to evaluate the reasons for attending in the transfer training.

#### Practice status

Practice status included clinical competence, whether transfer to/ add mental health practice registration, whether engage in mental / psychological work after training, the level of transfer training meeting job needs, the level of transfer training meeting theoretical learning needs, the level of transfer training meeting clinical practice needs, salary change compared with pre-training, the shame change compared with pre-training, whether join in continuing education after training and whether wanted to join in continuing education after training. The clinical competence was measured by Numeric Rating Scale, a ranking from 0 to 10 was used to indicate the level of clinical competence.

#### Workplace

Whether change workplace after training, whether the workplace before training has a mental / psychological department, whether the workplace after training has a mental/psychological department, institutional nature, institutional level, institutional property, and institutional affiliation were investigated in this part.

### Statistical methods

Data downloaded from Questionnaire Star in SPSS type data. All statistics analysis was performed by SPSS 22.0. A *P*<0.05 was considered statistically significant. Descriptive statistics was performed by mean ± standard deviation (SD), frequency and constituent ratio as appropriate. Independent sample T-test, one-way ANOVA were used to examine the clinical competence of physicians with different categorical variables. Spearman rank correlation analysis was used to assess the relationship between continuous data and clinical competence. A multiple linear regression analysis was conducted to explore the associated factors of clinical competence of physicians.

## Results

### Demographic

Demographic characteristics of participants and the clinical competence in different groups were presented in Table 1. The mean age of participants was (42.21 ± 7.96), about 70% (*n* = 177) of them were men. Most (*n* = 239, 82.1%) participants were practicing physician, and about 70% (*n* = 200) work in clinical medicine, 90% (*n* = 269) were intermediate ranks and titles and below. About three-quarter (*n* = 216, 74.2%) of all doctors were volunteered to attend the transfer training because of interests or job-demand.

**Table 1:** Demographic characteristics of scope-of-practice transfer psychiatrists and univariate analysis of clinical competence (*N* = 291)

Characteristics	Mean (SD)	N (%)	Clinical competence		
			Mean (SD)	Test statistics	*P*-value
Age (years)	42.21 ± 7.96			*r* = −0.087	0.137
Gender					
Male		177 (60.8)	8.13 ± 1.351	*T* = 1.491	0.137
Female		114 (39.2)	7.86 ± 1.645		
Practice level					
Practicing physician		239 (82.1)	8.00 ± 1.519	*T* = -0.619	0.536
Physician assistant		52 (17.9)	8.14 ± 1.268		
Practice category					
Clinical medicine		200 (68.7)	7.86 ± 1.450	*T* = -2.771	0.006
Traditional Chinese medicine		91 (31.3)	8.38 ± 1.480		
Professional ranks and titles					
Resident physician		180 (61.9)	8.08 ± 0.111	*F* = 0.544	0.581
Attending physician		89 (30.6)	7.89 ± 0.159		
Associate chief physician and above		22 (7.6)	8.14 ± 0.289		
Reasons for attending in the transfer training for psychiatrists					
Be interested and volunteered		193 (66.3)	8.39 ± 0.097	*F* = 21.023	<0.001
Job-demanded and volunteered		23 (7.9)	7.74 ± 0.211		
Hospital arranged		75 (25.8)	7.18 ± 0.179		

*Abbreviations:* *SD* standard deviation

### Practice status

Practice status of participants and the clinical competence in different groups were presented in Table 2. The clinical competence score was (8.02 ± 1.48). Most of participants transferred to mental health practice registration or add mental health practice registration (*n* = 220, 75.6%), and most of them engaged in mental / psychological work after transfer training (*n* = 202, 69.4%). Mean score of the level of transfer training meeting job needs, theoretical learning needs and clinical practice needs were (8.59 ± 1.62), (8.45 ± 1.74) and (8.48 ± 1.72) respectively. About 70% participants have a decrease of shame (*n* = 216, 74.2%), and the salary remained unchanged (*n* = 200, 68.7%) compared with pre-training.

**Table 2:** Practice status of scope-of-practice transfer psychiatrists and univariate analysis of clinical competence (*N* = 291)

Characteristics	Mean (SD)	N (%)	Clinical competence		
			Mean (SD)	Test statistics	*P*-value
Clinical competence	8.02 ± 1.48				
Whether transfer to/ add mental health practice registration					
Yes		220 (75.6)	8.19 ± 1.473	*T* = 3.398	0.001
No		71 (24.4)	7.52 ± 1.375		
Whether engage in mental / psychological work after training					
Yes		202 (69.4)	8.35 ± 1.410	*T* = 5.961	<0.001
No		89 (30.6)	7.29 ± 1.361		
The level of transfer training meeting job needs	8.59 ± 1.62			*r* = 0.525	<0.001
The level of transfer training meeting theoretical learning needs	8.45 ± 1.74			*r* = 0.470	<0.001
The level of transfer training meeting clinical practice needs	8.48 ± 1.72			*r* = 0.514	<0.001
Salary change compared with pre- training					
Increase		67 (23.0)	8.52 ± 0.164	*F* = 6.759	0.001
The same		200 (68.7)	7.93 ± 0.104		
Decrease		24 (8.2)	7.38 ± 0.312		
The shame change compared with pre- training					
Increase		6 (2.1)	8.34 ± 0.421	*F* = 0.488	0.614
The same		69 (23.7)	7.89 ± 0.176		
Decrease		216 (74.2)	8.06 ± 0.102		
Whether join in continuing education after training					
Yes		16 (5.5)	8.81 ± 1.378	*T* = 2.218	0.027
No		275 (94.5)	7.98 ± 1.471		
Whether wanted to join in continuing education after training					
Yes		161 (55.3)	8.37 ± 1.335	*T* = 4.588	<0.001
No		130 (44.7)	7.60 ± 1.535		

*Abbreviations:* *SD* standard deviation

### Workplace

Workplace of participants and the clinical competence in different groups were presented in Table 3. 34 (11.7%) participants changed their workplace after training, and it was 46 (15.8%) more workplaces having a mental / psychological department than pre-training. After training, about 30% (*n* = 89) physicians work at psychiatric hospital, 55% (*n* = 161) work at general hospital, most of them were public hospitals (*n* = 249, 85.6%) and affiliate to health system (*n* = 244, 83.8%).

**Table 3:** Workplace of scope-of-practice transfer psychiatrists and univariate analysis of clinical competence (*N* = 291)

Characteristics	N (%)	Clinical competence		
		Mean (SD)	Test statistics	*P*-value
Whether change workplace after training				
Yes	34 (11.7)	8.24 ± 1.706	*T* = 0.898	0.370
No	257 (88.3)	8.00 ± 1.444		
Whether the workplace before training has a mental / psychological department				
Yes	156 (53.6)	8.55 ± 1.335	*T* = 7.109	<0.001
No	135 (46.4)	7.41 ± 1.397		
Whether the workplace after training has a mental / psychological department				
Yes	202 (69.4)	8.30 ± 1.415	*T* = 5.052	<0.001
No	89 (30.6)	7.39 ± 1.422		
Institutional nature				
Psychiatric hospital	89 (30.6)	8.87 ± 0.118	*F* = 24.883	<0.001
General hospital	161 (55.3)	7.60 ± 0.121		
Primary medical and health care institution	41 (14.1)	7.86 ± 0.187		
Institutional level				
Tertiary	45 (15.5)	8.40 ± 0.243	*F* = 2.657	0.049
Secondary	130 (44.7)	8.04 ± 0.136		
Primary	57 (19.6)	7.60 ± 0.173		
No level	59 (20.3)	8.11 ± 0.163		
Institutional property				
Public	249 (85.6)	7.99 ± 0.096	*F* = 0.890	0.412
Private	36 (12.4)	8.31 ± 0.210		
Other	6 (2.1)	7.68 ± 0.424		
Institutional affiliation				
Health system	244 (83.8)	7.99 ± 0.096	8.174	<0.001
Prison system	17 (5.8)	7.12 ± 0.309		
Other	30 (10.3)	8.84 ± 0.159		

*Abbreviations:* *SD* standard deviation

### Relationships between study variables and clinical competence

The relationships between clinical competence and variables are presented in Tables 1, 2 and 3. The results of inter-group comparison and Spearman correlation analysis showed that practice category (*T* = -2.771, *P* = 0.006), reasons for attending in the transfer training for psychiatrists (*F* = 21.023, *P*<0.001), whether transfer to/ add mental health practice registration (*T* = 3.398, *P*<0.001), whether engage in mental / psychological work after training (*T* = 5.961, *P*<0.001), the level of transfer training meeting job needs (*r* = 0.525, *P*<0.001), the level of transfer training meeting theoretical learning needs (*r* = 0.470, *P*<0.001), the level of transfer training meeting clinical practice needs (*r* = 0.514, *P*<0.001), salary change compared with pre-training (*F* = 6.759, *P* = 0.001), whether join in continuing education after training (*T* = 2.218, *P* = 0.027), whether wanted to join in continuing educat3ion after training (*T* = 4.588, *P*<0.001), whether the workplace before training has a mental / psychological department (*T* = 7.109, *P*<0.001), whether the workplace after training has a mental / psychological department (*T* = 5.052, *P*<0.001), institutional nature (*F* = 24.883, *P*<0.001), institutional level (*F* = 2.657, *P* = 0.049), institutional affiliation (*F* = 8.174, *P*<0.001) were significantly associated with clinical competence among physicians.

### Regression analyses of clinical competence

Table 4 outlined the result of multiple linear regression model examining the relationships between study variables and clinical competence. Higher level of transfer training meeting job needs (*B* = 0.251, *P* = 0.002, 95% CI: 0.090 ~ 0.413), higher level of transfer training meeting clinical practice needs (*B* = 0.181, *P* = 0.018, 95% CI: 0.032 ~ 0.330) had statistically significant positive associations with clinical competence.

**Table 4:** Multiple linear regression on job competence of scope-of-practice transfer psychiatrists (*N* = 291)

Parameters	Clinical competence				
	B	SEE	β	95% CI	*P*-value
Constant	7.146	0.735		(5.699 ~ 8.593)	<0.001
The level of transfer training meeting job needs	0.251	0.082	0.275	(0.090 ~ 0.413)	0.002
Whether the workplace before training has a mental / psychological department	-0.667	0.154	−0.226	(−0.971 ~ − 0.364)	<0.001
Whether wanted to join in continuing education after training	−0.332	0.136	−0.112	(− 0.600 ~ − 0.064)	0.016
The level of transfer training meeting clinical practice needs	0.181	0.076	0.211	(0.032 ~ 0.330)	0.018
Whether join in continuing education after training	−0.538	0.288	−0.083	(−1.106 ~ 0.030)	0.063
Institutional nature (ref:psychiatric hospital)					
General hospital	−0.493	0.168	−0.166	(−0.824 ~ − 0.163)	0.004
primary medical and health care institution	−0.277	0.239	−0.065	(− 0.747 ~ 0.193)	0.247

*Abbreviations:* *SEE* standard error of estimation, *CI* confidence interval

We also found that the workplace before training without a mental / psychological department (*B* = -0.667, *P*<0.001, 95% CI:-0.971 ~ −0.364), participants who reluctant to join in continuing education after training (*B* = -0.332, *P* = 0.016, 95% CI:-0.600 ~ − 0.064), working at general hospital (*B* = -0.493, *P* = 0.004, 95% CI:-0.824 ~ − 0.163) got a lower clinical competence score.

## Discussion

### Whether the workplace before training has a mental / psychological department

We found that the physicians who worked at the workplace which has a mental / psychological department before training had better clinical competence. One possible reason for our result is that these physicians may have known some psychiatric knowledge which allows them to learn and absorb faster during training. Another possible reason of our result may be that the workplace which already have mental / psychological department can provide more support for physicians due to their organizational structure. Considering 85 counties didn’t have any mental health workforces and services in Sichuan province before the transfer training launched in 2016, physicians from these areas can only work alone without obtaining any help from other psychiatrics and department. We have found that only 34 (11.7%) physicians change their workplace after training, which means that most physicians’ work at the same place. Physicians from these workplaces without mental/psychological department have limit knowledge about psychiatry and psychology and may have worse working conditions and working foundation when they finished training.

### Whether wanted to join in continuing education after training

We also observed that the participants who wanted to join in continuing education after training had better clinical competence. One possible explanation of the result may be that physicians who wanted to join in continuing education after training are more interested in mental/ psychological knowledge, and they learned more about it than those who take little interest in their daily work and study.

Another possible reason may be that people wanted to join in continuing education have greater demand of related knowledge. 80.7% participants of who wanted to join in continuing education engaged in mental / psychological work after training, when only 55.4% participants of who didn’t want to join in continuing education engaged in related work. Their job responsibility could push them to learn related knowledge to be more competent.

### Institutional nature

Our study showed that participants worked at psychiatric hospital had better clinical competence than who worked at general hospital. Before the Transfer Training for psychiatrists Program launched in Sichuan province，there were 85 counties out of 183 counties couldn’t provide mental/psychological service, which means that these counties have no psychiatric hospitals/departments. Only 15 counties remain no mental/psychological service after 7 periods of training programs. Part of physicians from transfer training should establish mental/psychological branches by themselves. They may need more management, communication skills rather than mental and psychological knowledge, leading a relatively lower work competence. Another possible explanation maybe physician who works at psychiatric hospital can get potential support from department and other psychiatrists, making them better adapt to their clinical work.

### The level of transfer training meeting participants’ job and clinical practice demands

We also found that the higher level of transfer training meeting participants’ job and clinical practice needs, the higher clinical competence they have. Possible reason maybe higher demands mean participants had more motivation to learn, and what they learned match with their clinical practice. Thus, the continuing support, including theoretical knowledge, clinical skills, solution of practical problems, et al., is essential to improve the clinical competence of transfer psychiatrists. The online continuing education for scope-of-practice transfer psychiatrists is organized and launched in February this year, aiming to provide continuous support for them.

There were several limitations of this study that should be acknowledged. A major limitation was that there are significant gaps with respect to valid and pragmatic assessment tools to measure these clinical competencies. A self-made numeric rating scale developed by researchers was used in this study, which may lead to measurement error. We believe that this assessment gap may now be filled once the outcomes (competencies) have been better defined. The data collection was performed with non-random sampling method - convenience sampling. Our sample selection strategy could limit the generalizability of our results as it may weaken our sample’s representativeness. Nonetheless, the sample is adequately proportioned to the target group - physicians completing the transfer training and obtaining certificates, and with a response rate at 62.18%. This may bring about a more acceptable result. Cross-sectional studies cannot establish direction of causality, and all reported findings are of potential hazard, rather than actual risk for clinical competency. Risk factors as reported in this study, or in any cross-sectional study, should be considered as potential rather than actual as confirmation of actual risk can only be accurately established using prospective study designs. Though two main confounding factors had been considered and wrote into exclusion criteria to avoid bias in this study. There still have some potential confounding factors not considered for a lack of related references.

## Conclusion

The study showed that the clinical competence of scope-of-practice transfer psychiatrists needs to be improved. Whether workplace has mental/psychological departments was an important factor of clinical competence. Besides, interest of physicians is another crucial factor for their clinical competence. The continuing education of those psychiatrists may be one effective measure to improve their competency considering their factual working conditions.

## Data Availability

Data are available on reasonable request from the corresponding author.

## Abbreviations

CMHS: China Mental Health Survey
SD: standard deviation
ANOVA: Analysis of variance
SEE: standard error of estimation
CI: confidence interval
